# Two Filters for Acquiring the Profiles from Images Obtained from Weak-Light Background, Fluorescence Microscope, Transmission Electron Microscope, and Near-Infrared Camera

**DOI:** 10.3390/s23136207

**Published:** 2023-07-06

**Authors:** Yinghui Huang, Ruoxi Yang, Xin Geng, Zongan Li, Ye Wu

**Affiliations:** 1 School of Information Science and Technology, Nantong University, Nantong 226019, China; hyh9688@ntu.edu.cn; 2 School of Electrical and Automation Engineering, Nanjing Normal University, Nanjing 210046, China; yrx18132097823@163.com; 3 College of Building Environment Engineering, Zhengzhou University of Light Industry, Zhengzhou 450000, China

**Keywords:** near-infrared imaging, profile extraction, Gabor wavelets, watershed algorithm, matched filter

## Abstract

Extracting the profiles of images is critical because it can bring simplified description and draw special attention to particular areas in the images. In our work, we designed two filters via the exponential and hypotenuse functions for profile extraction. Their ability to extract the profiles from the images obtained from weak-light conditions, fluorescence microscopes, transmission electron microscopes, and near-infrared cameras is proven. Moreover, they can be used to extract the nesting structures in the images. Furthermore, their performance in extracting images degraded by Gaussian noise is evaluated. We used Gaussian white noise with a mean value of 0.9 to create very noisy images. These filters are effective for extracting the edge morphology in the noisy images. For the purpose of a comparative study, we used several well-known filters to process these noisy images, including the filter based on Gabor wavelet, the filter based on the watershed algorithm, and the matched filter, the performances of which in profile extraction are either comparable or not effective when dealing with extensively noisy images. Our filters have shown the potential for use in the field of pattern recognition and object tracking.

## 1. Introduction

Extracting the profiles of images is important in the field of image enhancement. It is critical in image division and target identification. It can provide information on key areas and the shape of targets. At the same time, it can be used to eliminate insignificant information. This is significant in this age of the metaverse, where professionals face challenges of processing the huge amounts of images stemming from industrial and personal applications.

Extracting profiles from images is a tough task, especially when the images contain rough noise and subjects in low contrast. Traditional ways of profiles extraction include using the operators of Roberts, Prewitt, Sobel, Canny, Laplacian, and LoG. When we look back to these operators, we found that they show several disadvantages. For example, they can present wrong regions of the edge or lead to rough edges and corrupted profiles [1]. Several efficient methods have been developed to overcome these issues. One group used quantum computation to extract image profiles, where flexible representation of quantum is used to construct robust algorithms [2]. Another group created fuzzy methods for extracting image profiles [3], which are demonstrated to consume little electric power and are extremely accurate. Additionally, researchers have used the concept of neural networks to develop new ways of obtaining the edge of the image [4,5,6,7,8,9]. For instance, a spiking neuron has been used to developed methods of detecting infrared images [4]. The technique of convolutional neural networks is applied to find the target in the images [5].

Furthermore, mathematical functions are widely studied for image extraction. One group used the Hausdorff derivative for image edge extraction, which shows better performance than the Sobel/Canny operator [10]. Another group developed an effective method via cooperative game formulation [11]. Other groups used wavelets to highlight the areas of interest in the images [12,13,14]. It is interesting to find out that the wavelets can be combined with spatial filtering in order to analyze the subjects possessing interrupted features [15,16,17,18]. It should be noted that these modified functions can lead to clear and continuous morphologies. This inspires us to believe that creating special functions may be a good way of obtaining the boundary of the image.

We hypothesized that reliable functions can be created to extract the boundaries of figures even if they contain a lot of noise. This prompted us to consider modifying and combining several popular functions, with the goal of creating a unique framework for filtering the frequency domain brought by wavelets transform. Multiple parameters related to the phase or the magnitude of the special functions would be widely employed to perform an efficient extraction of the profiles.

In addition, this method will be tested in practical applications, where acquiring clear profiles from the images with high degradation or weak light is challenging. We also chose to process the images acquired from a fluorescence microscope and a transmission electron microscope. This can be useful for the daily operation of acquiring the images obtained from fluorescence microscopes and transmission electron microscopes, where extensive noise can exist due to the contamination of the samples or inappropriate defocusing of the light or electron beam.

To accomplish this, we herein report two types of special functions to extract the profiles in the images. It should be mentioned that our way of obtaining the extracted profiles is not created with the intention to challenge the foundation of the current methods of profile extraction. Indeed, they are only supplements to current approaches. They contain mathematical functions that can be adjustable, which make them very useful for dealing with noisy images.

In our search for effective functions, two special functions were obtained which can be used to eliminate noise and extract the image boundary simultaneously. The next section is about the methods of building two kinds of special-function-based filters. In Section 3, images are processed by these two filters. The boundaries of the images can be extracted successfully. Their performance is then compared with several popular filters. Their application in processing images obtained from weak-light environments, a fluorescence microscope and a transmission electron microscope are shown. Finally, we discuss the advantages of using these filters for processing the images. They are envisioned to play an important role in medical imaging and target identification.

## 2. Methods

A fluorescence microscope (manufacturer: OLYMPUS, Tokyo, Japan, model: CKX41) was used to acquire the fluorescence image. Raw TEM images were obtained using a JEOL 1400 TEM (120 kV) (Tokyo, Japan). A solid-state laser (manufacturer: Gainlaser, Shenzhen, China, model: TR-A-IR10) whose emitting wavelength is 1046 nm was used for irradiating samples. A near-infrared CMOS camera (manufacturer: Zhongweiaoke, Shenzhen, China, model: 1080p) was used for obtaining near-infrared images.

With the objective of extracting the profiles of the images, we begin to test several functions. It is tough to obtain correct profiles in noisy images. We wish to develop a framework that can be used for processing the image when Gaussian white noise of a very high mean value is applied. Furthermore, we seek those frameworks which can be useful for extracting profiles from images in different circumstances.

### 2.1. Building a Filter Based on Exponential Function

In the following, we build a filter based on the exponential function, which is called the Exp filter, to exact the profiles from the image. This filtering processing was started from wavelets decomposition. We applied the wavelets decomposition to the pixel values of the input image. This generated a set of coefficient values, which is called *u(m,n)*. Then, we used the following form of the exponential function to obtain the value of *x(m,n)*:

(1) $$x\left(m,n\right)={\left[a\times e^{\frac{d\left(u\left(m,n\right)-b\right)}{g}}\right]}^{\mathrm{nn}}$$

Here, *m*, *n*, *nn*, *a*, *d*, *b*, and *g* are constants. We used the values of *x(m,n)* for wavelets recreation and constructed a new image.

The detailed algorithm is shown in Algorithm 1.

**Algorithm 1.** The algorithm of the Exp filter1: An image in JPG format is used.2: The image is converted into a gray image.3: The gray image is resized to be 1440 × 2560.4: We perform the wavelet transform using the pixels value of the gray image and get the coefficient value of *u(m,n).*5: We calculate the new coefficient value *x(m,n)* using the equation of $x\left(m,n\right)={\left[a\times e^{\frac{d\left(u\left(m,n\right)-b\right)}{g}}\right]}^{\mathrm{nn}}$. *d*, *b*, *g*, and *nn* are all constants.6: We calculate the inverse of wavelets transform using the value of *x(m,n)* and get new pixels values of *xx(m,n)*.7: We show the processed image using the values of *xx(m,n)*.

### 2.2. Building a Filter Based on Hypotenuse Function

We designed a filter based on the hypotenuse function, which is called the Hypot filter, to extract the profiles of the image. Similarly, the wavelets decomposition was applied to process the pixel values of the image. After this, we obtained the coefficient value of *u(m,n)*. Then, we used the combination of the cotangent function, hyperbolic sine function, inverse hyperbolic secant function, sine function, inverse hyperbolic cosine function, hyperbolic tangent function, and hypotenuse functions in order to obtain the new coefficient value of *u_6_(m*,*n)*, whose calculation is shown as follows:

(2) $${u_{1}(m,n)=a}_{1}\times \cot\left(a_{2}\times u\left(m,n\right)+a_{3}\right)+a_{4}\times \sinh(a_{5}\times u\left(m,n\right)+a_{6})\;$$

(3) $$u_{2}\left(m,n\right)=a_{7}\times \mathrm{asech}(a_{8}\times u\left(m,n\right)+a_{9})$$

(4) $$u_{3}\left(m,n\right)=a_{10}\times \sin\left(u\left(m,n\right)+a_{12}\right)+{a_{13}\times \mathrm{acosh}(a}_{14}\times u\left(m,n\right)+a_{15})+a_{16}\times \tanh(u\left(m,n\right)+a_{17})$$

(5) $${u_{4}(m,n)=a}_{18}\times \tanh\left(a_{19}\times u\left(m,n\right)+a_{20}\right)$$

(6) $$u_{5}\left(m,n\right)={(a}_{21}\times \mathrm{hypot}[u_{1}\left(m,n\right),u_{2}\left(m,n\right)])\times (1+u_{4}(m,n))$$

(7) $${u_{6}\left(m,n\right)=[\frac{u_{5}\left(m,n\right)}{u_{3}\left(m,n\right)}]}^{a_{22}}$$

Here, *a*_1_, *a*_2_, *a*_3_, *a*_4_, *a*_5_, *a*_6_, *a*_7_, *a*_8_, *a*_9_, *a*_10_, *a*_11_, *a*_12_, *a*_13_, *a*_14_, *a*_15_, *a*_16_, *a*_17_, *a*_18_, *a*_19_, *a*_20_, *a*_21_, and *a*_22_ are constants. cot means cotangent function. sinh stands for the hyperbolic sine function. asech represents the inverse hyperbolic secant function. sin means the sine function. acosh means the inverse hyperbolic cosine function. tanh stands for the hyperbolic tangent function. hypot means the hypotenuse function.

The value of *u*_6_*(m,n)* was used for the inverse of wavelet transform in order to make a new image. The algorithm is shown in Algorithm 2.

**Algorithm 2.** The algorithm of the Hypot filter1: An image in JPG format is used.2: We convert the image into a gray image.3: We perform wavelet transform using the pixels value of the gray image and get the coefficient value of *u(m,n)*.4: We calculate the new coefficient value *u*_5_*(m,n)* using the following equations:

${u_{1}(m,n)=a}_{1}\times \cot\left(a_{2}\times u\left(m,n\right)+a_{3}\right)+a_{4}\times \sinh(a_{5}\times u\left(m,n\right)+a_{6})\;\;$



$u_{2}\left(m,n\right)=a_{7}\times \mathrm{asech}(a_{8}\times u\left(m,n\right)+a_{9})\;u_{3}\left(m,n\right)=a_{10}\times \sin\left(u\left(m,n\right)+a_{12}\right)+$



${a_{13}\times \mathrm{acosh}(a}_{14}\times u\left(m,n\right)+a_{15})+a_{16}\times \tanh(u\left(m,n\right)+a_{17})$



${u_{4}(m,n)=a}_{18}\times \tanh\left(a_{19}\times u\left(m,n\right)+a_{20}\right)$



$u_{5}\left(m,n\right)={(a}_{21}\times \mathrm{hypot}[u_{1}\left(m,n\right),u_{2}\left(m,n\right)])\times (1+u_{4}(m,n))\;{u_{6}\left(m,n\right)=[\frac{u_{5}\left(m,n\right)}{u_{3}\left(m,n\right)}]}^{a_{22}}.$

5: We calculate the inverse of wavelet transform using the value of *u*_6_*(m,n)* and get the new pixels values of *yy(m,n)*.6: We show the processed image using the values of *yy(m,n)*.

## 3. Results

We will use our filters to process the degraded image and the image acquired from weak-light sources, a fluorescence microscope, and a near-infrared camera.

### 3.1. Processing the Images with High Degradation by Gaussian Noise

**Figure 1:**
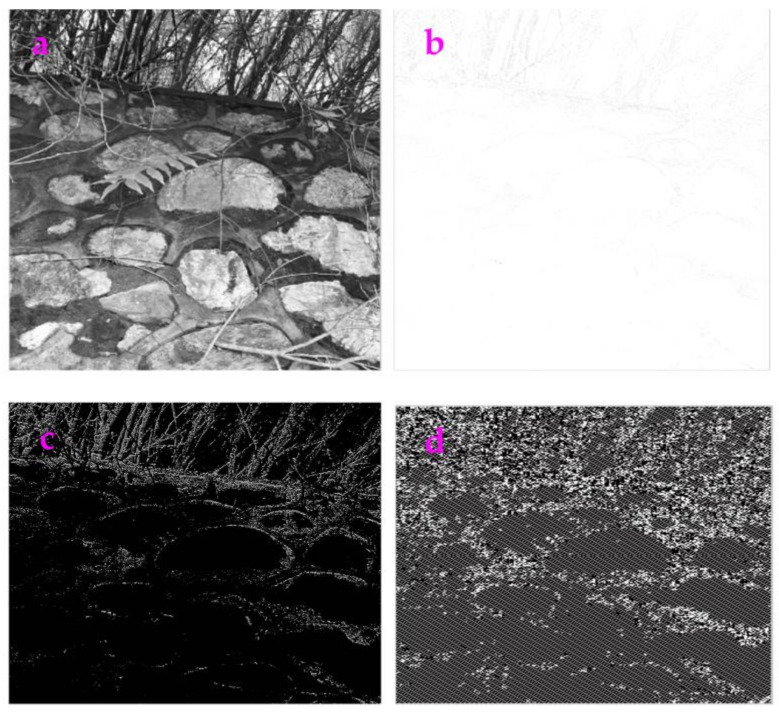
(**a**) An image called Rock. (**b**) The image Rock filled with high Gaussian noise (the mean value is 0.9). (**c**) We used the Exp filter to process the noisy image and obtain a clear profile. (**d**) We used the Hypot filter to process the noisy image and obtain a clear profile.

As shown in Figure 1a,b, we added Gaussian noise to the image of Rock and made a degraded figure. The Exp and Hypot filters can be used to extract a clear profile from this degraded figure. The circle-like profiles representing rocks can be seen.

In order to evaluate the effect of the enhancement for the image, we used the values of “measurement by entropy (*ME*)” and “Michelson contrast” [19]. *ME* can be obtained using the minimum (*m*_1_) and maximum (*m*_2_) values of the intensity in every block of the image:

(8) $$U_{i}=20\times \log\left(\frac{m_{2}}{m_{1}}\right)$$

(9) $$\mathrm{ME}=(\sum_{i=1}^{N}U_{i})/n$$

The Michelson contrast (*MC*) can be calculated as follows:

(10) $$\mathrm{MC}=(m_{3}-m_{4})/(m_{3}+m_{4})$$

Here, *m*_3_ is the highest intensity value of the image. *m*_4_ is the lowest intensity value of the image.

The value of *ME* and *MC* for the image Rock is shown in Table 1. We can see that the *ME* value of the Exp filter is smaller than that of the Hypot filter. This indicates that the filtering impact of the Hypot filter is higher than that of the Exp filter. The *MC* value for these two filters is equal.

### 3.2. Processing the Weak-Light Images

**Figure 2:**
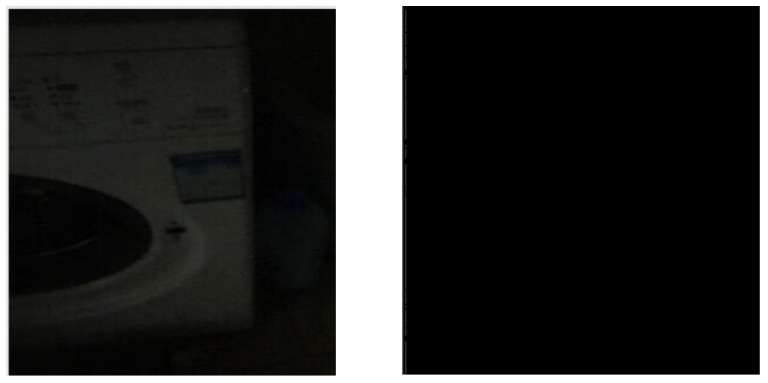
(**Left**) A weak-light image called Washing Machine. (**Right**) Image processed by the Sobel operator.

As shown in Figure 2, after we used the Sobel operator to process the weak-light image, it was difficult to find any detailed profile in the image. Here, the Sobel operator is a well-known differential operator that is generally used to obtain the edge profiles of any ordinal image [20]. It can hold the following form:

(11) $$\frac{\partial f(x,y)}{\partial x}\approx \frac{f\left(x+1,y+1\right)-f(x-1,y+1)}{2}$$

(12) $$\frac{\partial f(x,y)}{\partial y}\approx \frac{f\left(x+1,y+1\right)-f(x+1,y-1)}{2}$$

where *f(x,y)* is the grayscale value of the image.

**Figure 3:**
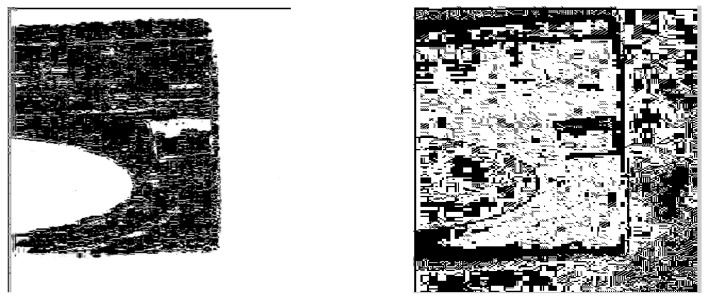
(**Left**) The image of Washing Machine processed by the Exp filter. (**Right**) The image of Washing Machine processed by the Hypot filter.

When we used the Exp and Hypot filters, we are able to obtain the shape of the washing machine (Figure 3). We calculated the value of the *ME* and *MC* of the filters for the image Washing Machine (Table 2). The *ME* value of the Hypot filter is higher than that of the Exp filter, which illustrates that the filtering impact of the Hypot filter is higher than that of the Exp filter. For the Sobel operator, the values of *ME* and *MC* are calculated as 0 and 1.3173. An *ME* value of zero may indicate the poor performance of the Sobel operator.

### 3.3. Processing the Fluorescence Images

**Figure 4:**
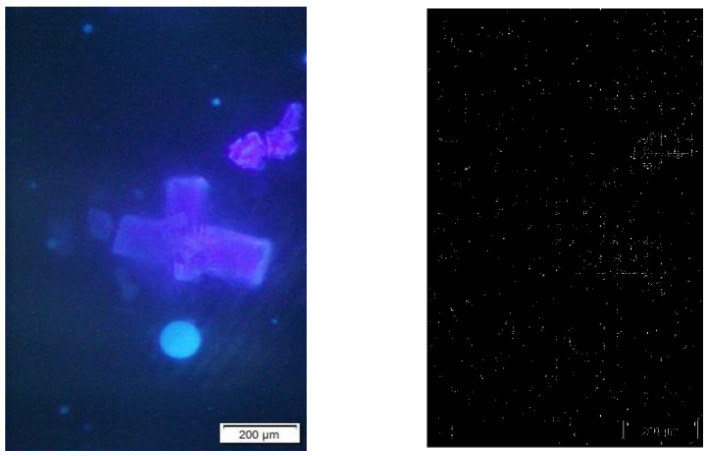
(**Left**) An image called p90. (**Right**) The image processed by Sobel operator.

**Figure 5:**
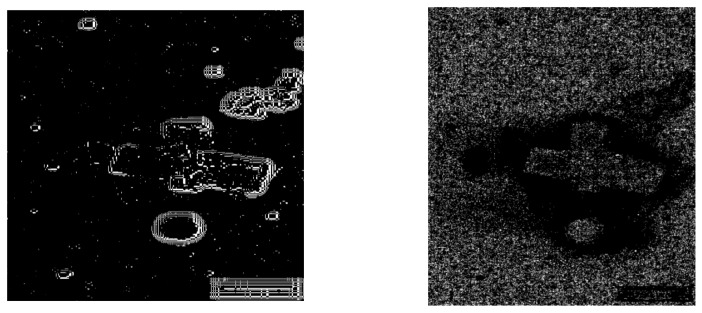
(**Left**) p90 processed by the Exp filter. (**Right**) p90 processed by the Hypot filter.

As shown in Figure 4, the profile in an image acquired from a fluorescence microscope was difficult to obtain via the Sobel operator. However, if the Exp filter and the Hypot filter were used, they were able to extract the profile (Figure 5).

When we use the *ME* and *MC* values to evaluate the performance of the Exp filter, the Hypot filter and the Sobel operator for the image p90 (Table 3), we can see that the Hypot filter obtains the highest value of *ME* and the lowest value of *MC*.

### 3.4. Noise Helps to Extract the Profile from the Images Acquired from the Transmission Electron Microscope

**Figure 6:**
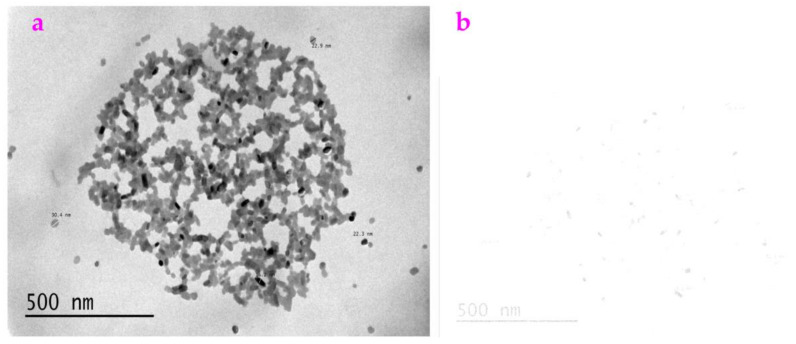

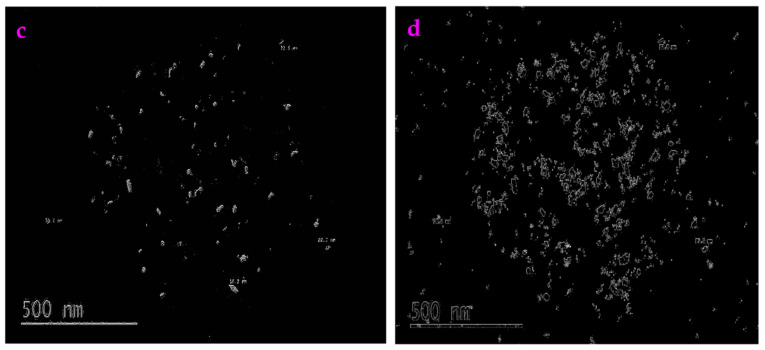
Noise is not harmful. (**a**) An image acquired from the transmission electron microscopy, which is called AgTi. (**b**) We added Gaussian noise with the mean value of 0.9 to the image of AgTi. (**c**) This noisy image was processed by the Exp filter. (**d**) This noisy image was processed by the Hypot filter. Here, it is clearly shown that the Exp filter and the Hypot filter can be used to extract the TEM images containing high noise.

**Figure 7:**
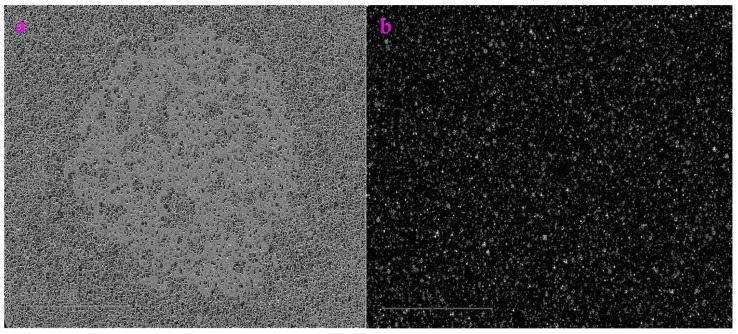
No noise was added. (**a**) The AgTi image processed by the Exp filter. (**b**) The AgTi image processed by the Hypot filter. Compared to those in Figure 6, it is barely possible to obtain the profiles.

In order to show that these filters we created are useful, we applied these filters to the images obtained from the transmission electron microscope (TEM). Figure 6a is a TEM image of nano-particles, which is called AgTi. These nanoparticles are synthesized by the reaction between a metal complex and an organic acid. The synthesized nanoparticles have the size of around 20–30 nm. As shown in Figure 6b, we added Gaussian noise with a mean value of 0.9 to the image of AgTi. When we used the Exp filter and the Hypot filter respectively to process Figure 6b, which contained Gaussian noise, we obtained a clear profile. However, when we used these filters to process Figure 6a, the profile was barely acquired. This indicates that adding Gaussian noise maybe helpful when trying to extract the TEM images using the Exp filter and the Hypot filter (Figure 7).

Using the results in Figure 6, we calculated the *ME* value and the *MC* value for the image AgTi (Table 4), which showed that the *ME* value of the Exp filter is smaller than that of the Hypot filter and that the *MC* value of the Exp filter is greater than that of the Hypot filter.

### 3.5. Comparisons to Several Well-Known Filters

**Figure 8:**
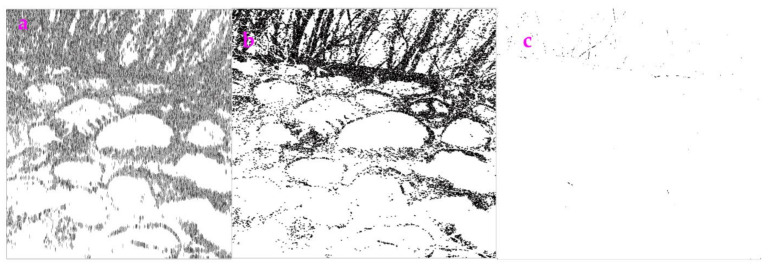
The image which contained the Gaussian white noise with the mean value of 0.9 was processed by several filters: (**a**) the filter based on Gabor wavelet, (**b**) the filter based on watershed algorithm, and (**c**) the matched filter.

**Figure 9:**
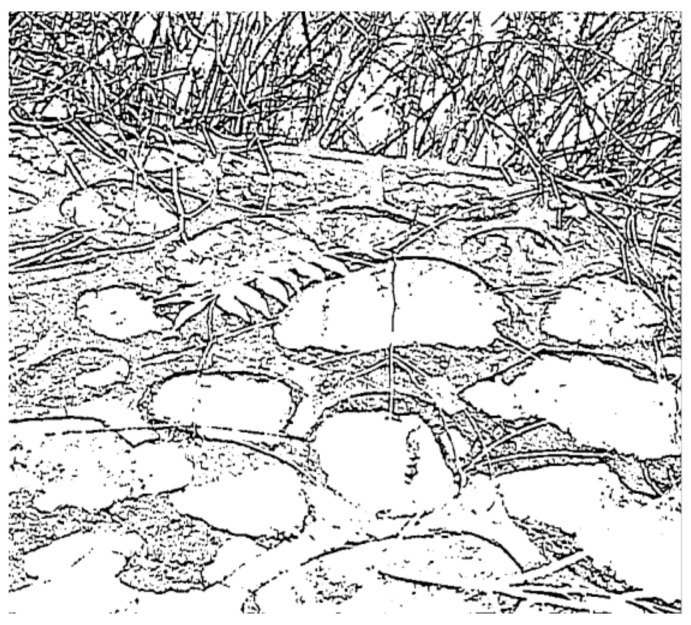
We reduced the Gaussian white noise. The mean value was set to 0.5. When it was processed by the matched filter, a clear profile could be extracted.

For the purpose of a comparative study, we used several well-known filters to process the noisy images, including the filter based on Gabor wavelet [21], the filter based on the watershed algorithm [22] and the matched filter [23]. The image of Rock was tested. Gaussian noise with a mean value of 0.9 was added in order to create an image with very high noise. With such high noise, profiles extracted via the filter based on Gabor wavelets and the filter based on the watershed algorithm are visible (Figure 8a,b). The profiles extracted from the matched filter were hard to be seen (Figure 8c). This may be due to the existence of extensive noise. Actually, if we reduced the mean value of the Gaussian noise to a value of 0.5, clear profiles could be extracted via the matched filter (Figure 9).

Using Figure 8a–c, we can calculate the *ME* values and the *MC* values of these three filters, which are listed in Table 5. When we compare the value for these filters, we can find that the filter based on the Gabor wavelet obtains the highest value of *ME* and the lowest value of *MC*.

Using Figure 9 with the match filter, we find that the calculated *ME* value and *MC* value are 2.3469 and 1.4506, respectively. Compared to the values in Table 5, it indicates that the increase in the Gaussian noise turns down the *ME* value and the *MC* value.

### 3.6. Processing the Images Acquired from the Near-Infrared Camera

**Figure 10:**
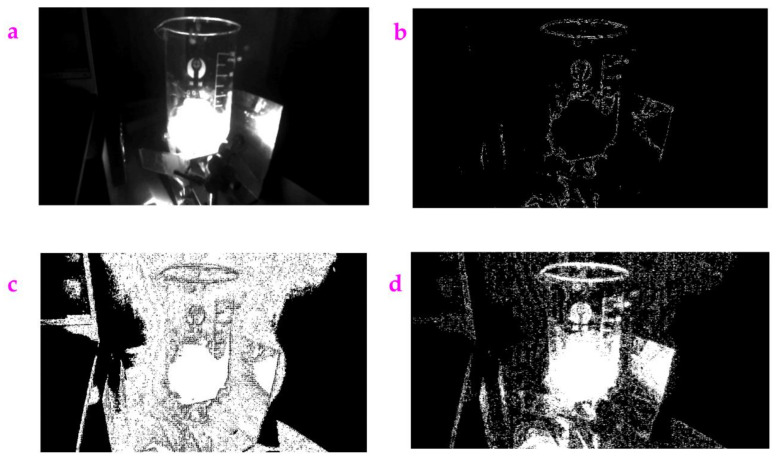
(**a**) An input image called NIR for processing. This image is acquired from near-infrared camera. (**b**) The profile acquired from the Sobel operator. (**c**) The profile acquired from the Exp filter. (**d**) The profile acquired from the Hypot filter.

The images obtained from the near-infrared imaging generally contain a lot of noise due to the emission of autofluorescence, low contrast, and little imaging depth [24]. This brings difficulty when processing these images. Figure 10a is an image acquired from the near-infrared camera. A glass beaker put above a bracket was surrounded by a Dell laptop and an iron sheet. Extracting its profile using the Sobel operator can result in a loss of details (Figure 10b). However, using the Exp filter or the Hypot filter, the detailed profile of the laptop hiding behind the bottle can be shown (Figure 10c,d). As we compared the *ME* value and the *MC* value with the Exp filter, the Hypot filter, and the Sobel operator for the NIR image (Table 6), it could be seen that the Exp filter holds the highest *ME* value and the highest *MC* value.

### 3.7. Tuning of the Filters

**Figure 11:**
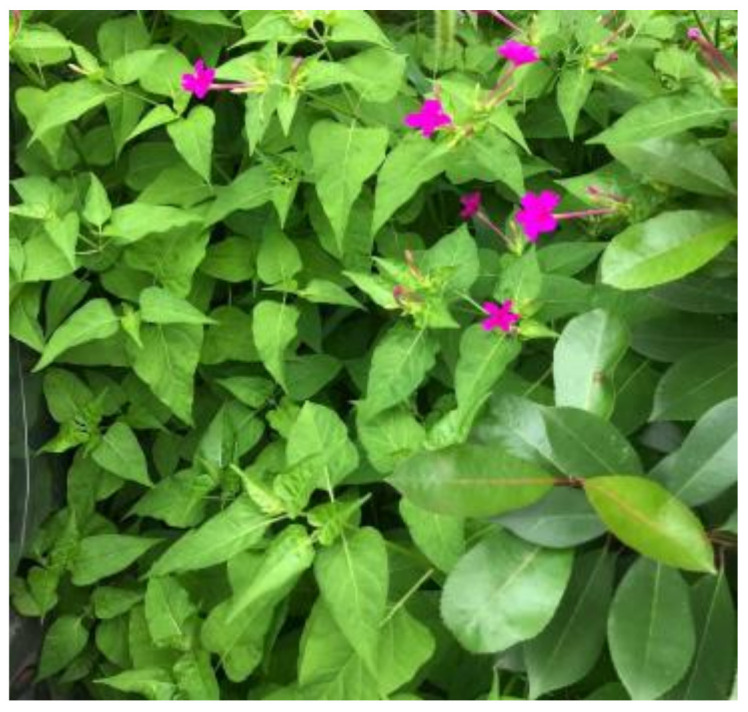
An image called *Mirabilis jalapa*.

Figure 11 is an image called *Mirabilis jalapa*, which will be processed by the Exp filter and the Hypot filter with different key parameters. This experiment will clearly show how the performance of the filters can be impacted. It should be noted that the tuning of the filters can be shown in some parameters, which we will show, and we discard those trivial effects.

**Figure 12:**
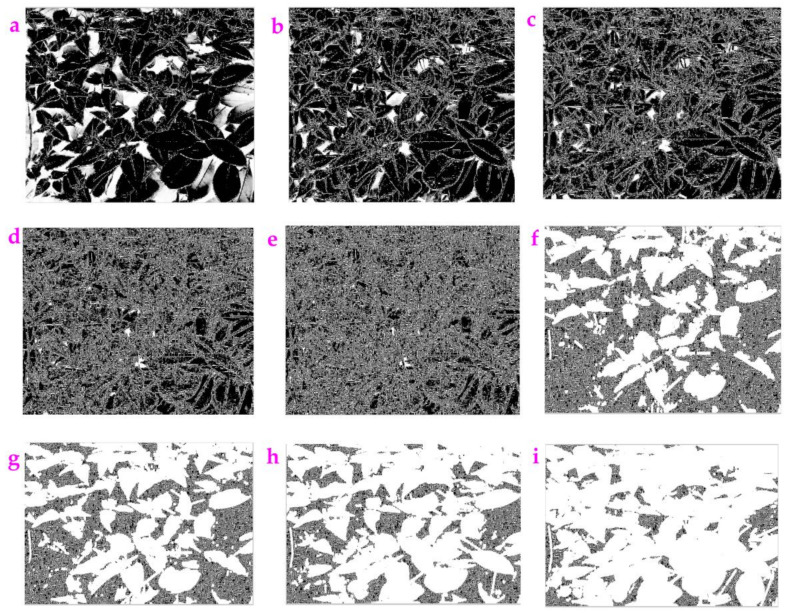
The image *Mirabilis jalapa* was processed by the Exp filter with different values of nn: (**a**) nn = 1, (**b**) nn = 2, (**c**) nn = 3, (**d**) nn = 6, (**e**) nn = 10, (**f**) nn = 44, (**g**) nn = 50, (**h**) nn = 60, (**i**) nn = 90.

As shown in Figure 12, the increase in the nn value will make the features become dark, then white. As shown in Table 7, the calculated *ME* value shows the nonlinear trend, and the *MC* value stays at 1 with the increasing nn value.

**Figure 13:**
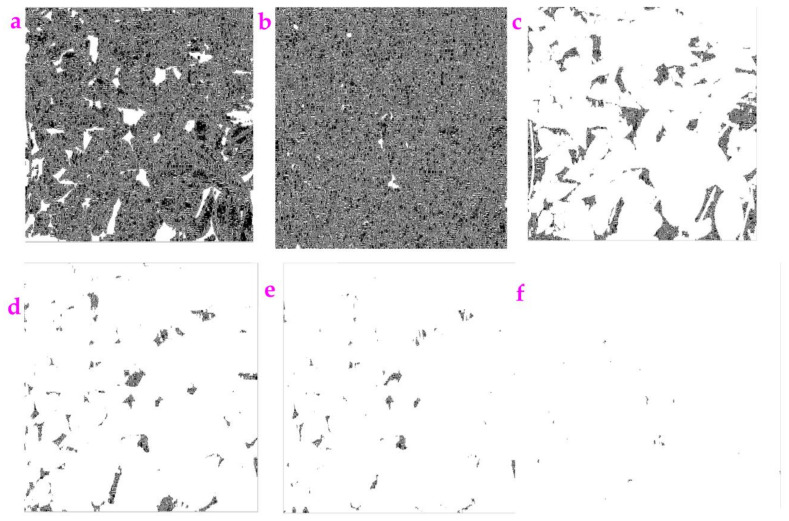
The image *Mirabilis jalapa* was processed by the Exp filter with different values of d: (**a**) d = 0.0078, (**b**) d = 0.0178, (**c**) d = 0.0578, (**d**) d = 0.0878, (**e**) d = 0.1178, (**f**) d = 0.2178.

We changed the value of d and drew the images processed by the Exp filter (Figure 13). Clearly, the enhancement of the d value will blur the image and result in missing features. The calculated *ME* value and *MC* value are shown in Table 8. The *ME* value showed a nonlinear trend and the *MC* value keeps constant as the d value is increasing.

**Figure 14:**
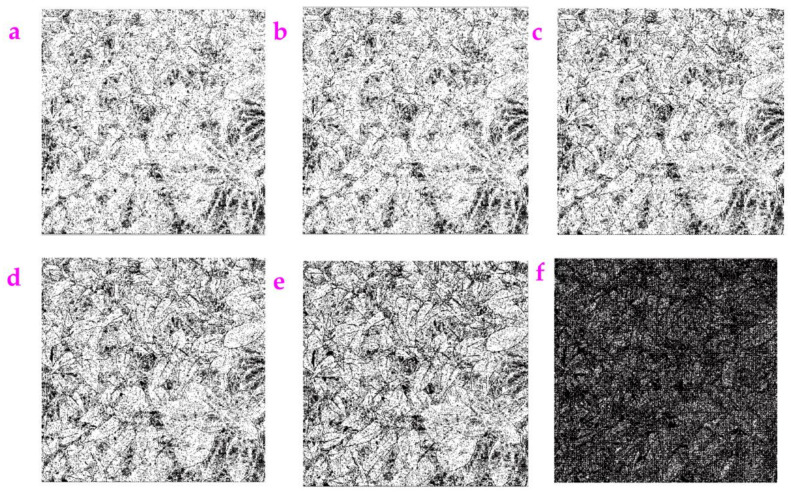
The image *Mirabilis jalapa* was processed by the Hypot filter with different values of *a*_18_: (**a**) *a*_18_ = 7, (**b**) *a*_18_ = 700, (**c**) *a*_18_ = 700,000, (**d**) *a*_18_ = 700,000,000, (**e**) *a*_18_ = 700,000,000,000, (**f**) *a*_18_ = 70,000,000,000,000,000.

**Figure 15:**
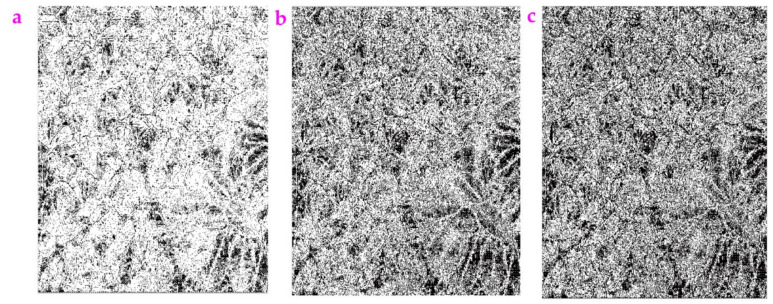

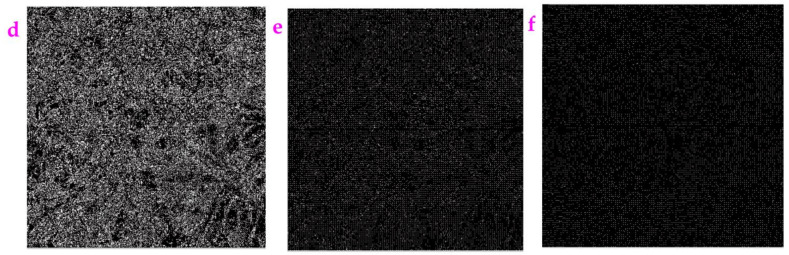
The image *Mirabilis jalapa* was processed by the Hypot filter with different values of *a*_21_: (**a**) *a*_21_ = 1, (**b**) *a*_21_ = 40,000, (**c**) *a*_21_ = 4,000,000,000, (**d**) *a*_21_ = 400,000,000,000,000, (**e**) *a*_21_ = 4,000,000,000,000,000, (**f**) *a*_21_ = 40,000,000,000,000,000.

**Figure 16:**
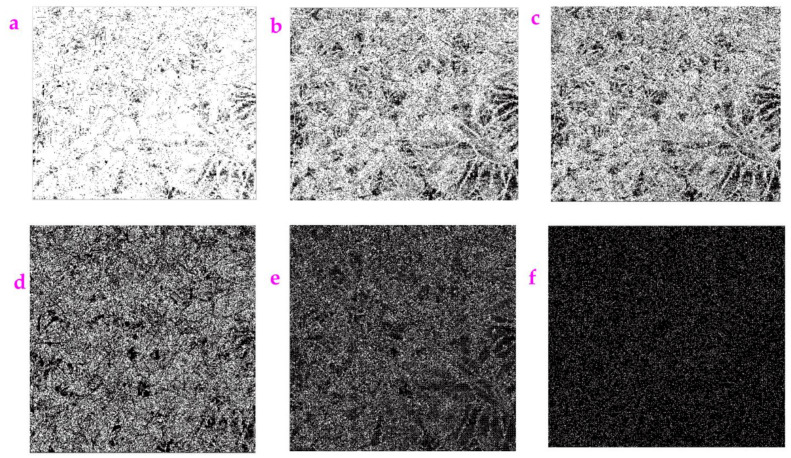
The image *Mirabilis jalapa* was processed by the Hypot filter with different values of *a*_22_: (**a**) *a*_22_ = 10, (**b**) *a*_22_ = 20, (**c**) *a*_22_ = 30, (**d**) *a*_22_ = 180, (**e**) *a*_22_ = 220, (**f**) *a*_22_ = 400.

Figure 14, Figure 15 and Figure 16 show the resulting images associated with the enhancement of the a_18_ value, the a_21_ value, and the a_22_ value, which leads to the appearance of dark sections in the images. The *ME* value and the *MC* value are listed in Table 9, Table 10 and Table 11. The *ME* value shows a nonlinear trend while the *MC* value stays at 1.

## 4. Limitations and Perspectives of Our Designed Filters

Our designed filters have shown their potential in extracting the profiles from images obtained in weak-light conditions and from fluorescence microscopes, transmission electron microscopes, and the near-infrared cameras. However, limitations can be seen in our designed filters, and improvement needs to be made in future research.

The parameters used in the filters have to be tailored in order to fit different images. One of our future projects may be focused on developing adaptive frameworks that can be applied to different images.

Moreover, the exact mechanism allowing these filters to effectively extract the image profiles needs to be explored in our future study.

In addition, the generated images from our frameworks are gray, which lack vivid and direct information. Our future work may be focused on developing a method that can be processed to generate colored images.

In classical image processing, many operators have been developed or modified in order to adapt to the current vast demand of information in industry and research. These classical operators hold great merit, and their performance is incredible. Our work shows two additional solutions to these existing methods. One merit of our work is that our solution is based on the frameworks associated with functions. The scales of the functions can be adjusted in order to meet a particular requirement. Furthermore, the technique of the profile extraction brings a much greater chance to meet a wider set of requirements for quality control in industry, where the checking for product defects plays a key role in obtaining a profit. In addition, our filters can be used to extract profiles from images degraded by Gaussian noise. This may have applications in cryptography, where people may cover the clandestine image profiles in the transfer of information and use our method to restore the profiles when receiving the information.

Another valuable merit of our methodology would be the use of wavelets in our framework. This makes our frameworks more dynamic since many wavelets can be flexibly adjusted, including Haar, Daubechies, Biorthogonal, Coiflet, SymletsA, Morlet, MexicanHat, and Meyer. These wavelets can be modified using the technique of wavelets uplifting, which brings a huge set of special functionalities, including image enhancement, image compression, object tracking, pattern identification, image smoothing, image sharpening, image denoising, image blunting, and image fusion.

## 5. Conclusions

Extracting a profile from an image has always been a key subject in image identification. Although many algorithms have been created, special-function-based filters are still a subject that is in its infancy stage. Our work presents a useful framework for profile extraction via two kinds of filters. We built two filters based on the exponential function and the hypotenuse function. They have shown a good ability to extract profiles from a noisy image. The edge profile can be extracted even when Gaussian white noise with mean value of 0.9 is added to the original images. They can be also useful for highlighting the images acquired using weak light sources, fluorescence microscopes, transmission electron microscopes, and the near-infrared cameras. Our methods may have potential application in medical imaging and subject identification, where profile extraction is an important task.

## Figures and Tables

**Table 1:** Evaluation of the filters for the image Rock.

Filters	*ME*	Michelson Contrast
Exp	0.0217	1
Hypot	34.3448	1

**Table 2:** Evaluation of the filters for the image Washing Machine.

Filters/Operator	*ME*	Michelson Contrast
Exp	1.1673	1
Hypot	19.4937	1
Sobel	0	1.3173

**Table 3:** Evaluation of the filters for the image p90.

Filters	*ME*	Michelson Contrast
Exp	0.0082	1.3315
Hypot	37.2042	1
Sobel	0	1.3125

**Table 4:** Evaluation of the filters for the image AgTi.

Filters	*ME*	Michelson Contrast
Exp	0.059	1.3064
Hypot	0.1123	1

**Table 5:** Evaluation of other filters for the image p90.

Filters	*ME*	Michelson Contrast
The filter based on the Gabor wavelet	15.5230	1.2699
The filter based on watershed algorithm	1.5496	1.3535
The match filter	0.0305	1.4255

**Table 6:** Evaluation of the filter for the NIR image.

Filters	*ME*	Michelson Contrast
Exp	31.6915	1.4324
Hypot	7.8975	1
Sobel	0	1.3411

**Table 7:** Evaluation of the Exp filter with different values of nn.

nn	*ME*	Michelson Contrast
1	2.1461	1
2	0.7534	1
3	0.4282	1
6	0.3502	1
44	5.1027	1
50	5.0971	1
60	4.8368	1
90	3.5409	1

**Table 8:** Evaluation of the Exp filter with different values of d.

d	*ME*	Michelson Contrast
0.0078	2.1504	1
0.0178	2.0325	1
0.0578	3.0276	1
0.0878	1.5115	1
0.1178	0.8224	1
0.2178	0.0778	1

**Table 9:** Evaluation of the Hypot filter with different values of *a*_18_.

*a*_18_	*ME*	Michelson Contrast
7	26.3231	1
700	26.7869	1
700,000	27.5250	1
700,000,000	28.5303	1
700,000,000,000	29.2491	1
70,000,000,000,000,000	38.131	1

**Table 10:** Evaluation of the Hypot filter with different values of *a*_21_.

*a*_21_	*ME*	Michelson Contrast
1	19.9629	1
40,000	37.1081	1
4,000,000,000	37.4871	1
400,000,000,000,000	35.1057	1
4,000,000,000,000,000	27.8838	1
40,000,000,000,000,000	0.6647	1

**Table 11:** Evaluation of the Hypot filter with different values of *a*_22_.

*a*_22_	*ME*	Michelson Contrast
10	19.9629	1
20	30.6965	1
30	35.7034	1
180	37.6083	1
220	48.9824	1
400	20.9649	1

## Data Availability

Not applicable.
